# Real-world effectiveness of repeated intravenous ketamine infusions for treatment-resistant depression in transitional age youth

**DOI:** 10.1177/02698811231171531

**Published:** 2023-05-16

**Authors:** Noah Chisamore, Kevork Danayan, Nelson B Rodrigues, Joshua D Di Vincenzo, Shakila Meshkat, Zoe Doyle, Rodrigo Mansur, Lee Phan, Farhan Fancy, Edmond Chau, Aniqa Tabassum, Kevin Kratiuk, Anil Arekapudi, Roger S McIntyre, Joshua D. Rosenblat

**Affiliations:** 1 Mood Disorders Psychopharmacology Unit, University Health Network, Toronto, ON, Canada; 2 Brain and Cognition Discovery Foundation, Toronto, ON, Canada; 3 Canadian Rapid Treatment Centre of Excellence, Mississauga, ON, Canada

**Keywords:** Depression, ketamine, major depressive disorder, transitional age youth, treatment-resistant depression

## Abstract

**Background:**

Ketamine is an emerging treatment for treatment-resistant depression (TRD) associated with rapid and robust improvements in depressive symptoms and suicidality. However, the efficacy and safety of ketamine in transitional age youth (TAY; age 18–25) populations remains understudied.

**Methods:**

In this retrospective analysis, TAY patients (*n* = 52) receiving ketamine for TRD were matched for sex, primary diagnosis, baseline depression severity, and treatment resistance with a general adult (GA) sample (age 30–60). Patients received four ketamine infusions over 2 weeks (0.5–0.75 mg/kg over 40 min). The primary outcome was the change in Quick Inventory of Depressive Symptomatology Self-Report 16-item (QIDS-SR16) over time. Secondary outcomes were changes in QIDS-SR16 suicidal ideation (SI) item, anxiety (Generalized Anxiety Disorder 7-item (GAD-7)), and adverse effects (ClinicalTrials.gov: NCT04209296).

**Results:**

A significant main effect of infusions on reduction of total QIDS-SR16 (*p* < 0.001), QIDS-SR16 SI (*p* < 0.001), and GAD-7 (*p* < 0.001) scores was observed in the TAY group with moderate effect sizes, indicative of clinically significant improvements in depression, anxiety, and suicidality. There were no significant differences between TAY and GA groups on these measures over time, suggesting comparable improvements in both groups. Safety and tolerability outcomes were comparable between groups with only mild, transient adverse effects observed.

**Conclusion:**

Ketamine was associated with comparable clinical benefits, safety, and tolerability in a TAY sample as compared to a matched GA TRD sample.

## Introduction

A large proportion of adult patients (⩾18 years of age) with depressive disorders have an inadequate response to antidepressants. Treatment-resistant depression (TRD) is typically defined as depression that is not responsive to two or more adequate antidepressant trials (McIntyre et al., 2014; Thase and Rush, 1997). Approximately 30% of patients do not achieve remission from depressive symptoms when treated with monoaminergic antidepressants (Zhdanava et al., 2021). TRD is also responsible for a disproportionate amount of health care burden and cost related to depression, accounting for 47% of all costs relating to major depressive disorder (MDD) in the United States despite only 31% of MDD cases being classified as TRD (Zhdanava et al., 2021). Repeated and sequential nonresponses to antidepressant treatment are associated with an increased risk of suicidality, depression relapse, poor quality of life, worsening function, and all-cause mortality (Rush et al., 2006). Therefore, novel treatments specifically for TRD with substantially greater efficacy are urgently needed.

Transitional age youth (TAY), defined as those between the ages of 18–25 years, represent a unique population with distinct neurobiological differences compared to both pediatric (<18 years of age) and general adult (GA) (⩾25 years of age) populations (Chan et al., 2019). Between 18 and 25 years of age, brain development is still occurring, moving well beyond the boundary of pediatric psychiatric care set at 18 years of age (Johnson et al., 2009). TAY are at a greater risk for the emergence of mental illnesses, such as MDD and bipolar disorder (BD), and are more likely to endorse symptoms of suicidality than adult patients (Chan et al., 2019; Tyson and Robb, 2021). The age range between 18 and 25 years is crucial for developing a sense of independence, identity formation, social functioning, and achievements that may be particularly susceptible to development of depressive disorders (Tyson and Robb, 2021). Early onset depression presents a greater risk of depression relapse, worsening of symptoms, and comorbid substance use disorders compared to onset after the age of 25 (Martel, 2021). Moreover, with the potential risk of treatment-emergent suicidality with serotonergic antidepressants in patients under the age of 25, it is important to identify alternative treatment options that do not rely on monoaminergic mechanisms (Li et al., 2022). It should be acnowledged the existence of a link between monoaminergic antidepressants and suicidal ideation (SI) in younger patients is tentatively associative and a controversial debate (Murphy et al., 2021). Conventional augmentation options (i.e., second-generation antipsychotics) are also problematic in this age group with a risk of metabolic effects in younger patients (Krill and Kumra, 2014; Pillinger et al., 2020; Tu et al., 2019). It is unclear if these adverse effects occur at a greater rate in TAY than in GAs, although children are at a greater risk of metabolic effects of antipsychotics (Libowitz and Nurmi, 2021).

Ketamine is an *N*-methyl-d-aspartate receptor antagonist with rapid antidepressant effects supported by evidence from replicated clinical trials (McIntyre et al., 2020; Phillips et al., 2019; Singh et al., 2016) and real-world evidence (Alnefeesi et al., 2020; Di Vincenzo et al., 2021; Lipsitz et al., 2021; Serafini et al., 2014; Wilkinson et al., 2018). Of note, only intranasal S-ketamine is currently approved as an adjunctive treatment for TRD in adult patients by the Food and Drug Administration (Wilkinson et al., 2018). In contrast, IV ketamine is only approved as an anesthetic. While the safety and efficacy of IV ketamine for TRD in adults has been established in the literature, there is a paucity of data on its use specifically in the TAY population. To date, the literature has focused on the efficacy of IV ketamine for TRD in adolescents or across a range of ages so that TAY patients are not distinguished from other adults, despite the neurobiological and social differences between these groups (Di Vincenzo et al., 2021; Dwyer et al. 2021; Johnson et al., 2009). In the adolescent population (ages 13–17), IV ketamine was found to be effective in reducing depression symptoms over 2 weeks, while also being well-tolerated (Dwyer et al., 2021). This current retrospective analysis of real-world data seeks to discern whether TAY patients with TRD demonstrate an effective response to IV ketamine and whether the nature of this response is significantly different from GA patient populations. TAY patients will also be evaluated to determine if they experience a difference in tolerability and safety towards IV ketamine use regarding adverse side effects. We hypothesize that repeated ketamine infusions will be associated with comparable antidepressant effects and tolerability in TAY patients with TRD compared to the GA population.

Research on how ketamine may affect the brain in younger patients (⩽25 years old) is currently still limited to extrapolating animal model research on developing brains of rats (Kim et al., 2021). While neurotoxic effects were observed in the frontal cortex of developing rat brains in response to ketamine infusions, the effects appeared dependent on the dose and frequency of ketamine (Liu et al., 2011). Both the frequency and dose of ketamine used were significantly greater than those used for treating depression intravenously (Kim et al., 2021). Dose dependent increases in neurodegenerative and cognitive impairment were observed in daily ketamine doses ranging from 7.5 to 80 mg/kg (Kim et al., 2021; Onaolapo et al., 2019). However, even a dose of 7.5 mg/kg is ten times higher than the maximum dose used in this study, and was administered far more frequently at daily doses for 8 weeks. An increase in glutamate levels was also observed, similarly to the theorized mechanism for ketamine’s antidepressant effects in humans (Onaolapo et al., 2019). This suggests the antidepressant mechanism for ketamine is consistent across age populations, and while neurodegenerative effects can occur, they occur only in far more frequent and concentrated dosing than is ever used therapeutically in depression protocols.

## Methods

### Clinical protocol

This retrospective analysis presents data obtained from patients at the Canadian Rapid Treatment Center of Excellence (CRTCE), located in Mississauga, Ontario, Canada. The CRTCE is a community outpatient clinic that administers IV ketamine for patients with TRD. All patients treated at the CRTCE met the criteria for stage 2 TRD, defined as having an inadequate response to at least two adequate antidepressant trials (Thase and Rush, 1997). Patients were referred to the CRTCE by psychiatrists or primary care providers and all had a mood disorder as the primary diagnosis. Prior to beginning treatment, informed consent is obtained from patients regarding data collection and chart review for research purposes. Patients with a history of psychosis or current substance use disorders were excluded from receiving treatment. Patients with a substance use disorder were included if they were able to abstain from the given substance for 3 months prior to treatment. If eligible, participating patients were assessed by a staff psychiatrist to confirm the diagnosis and whether IV ketamine would be an appropriate treatment choice. Diagnoses were made in accordance with criteria provided in the Diagnostic and Statistical Manual Fifth Edition. Anesthesiologists reviewed patient medical records for the presence of an unstable medical condition such as tachycardia or uncontrolled hypertension.

Patients received four IV ketamine infusions over the course of 2 weeks. Infusions began with ketamine at 0.5 mg/kg of body weight diluted in a 0.9% saline solution. If after two infusions, patients still had an incomplete clinical response, then the dosage may be increased to 0.75 mg/kg provided the lower dose was well-tolerated.

The Quick Inventory of Depressive Symptomatology Self-Report 16-item (QIDS-SR16) was used to measure the severity of depressive symptoms. The QIDS-SR16 classifies depression severity in the last 7 days, with total scores ranging from no depression present (0–5) or very severe depression (21–27). The QIDS-SR16 was administered at baseline, after each subsequent infusion (1–4), and at post-treatment follow-up with a staff psychiatrist. An item from the QIDS-SR16 on SI was used to assess the severity and presence of suicidality in TAY patients with TRD and any changes in suicidality across baseline, post-infusions, and post-treatment follow-ups. The Generalized Anxiety Disorder 7-item (GAD-7) scale was used to assess generalized anxiety symptoms. The GAD-7 is a seven-item questionnaire that evaluates anxiety severity based on the number of days affected out of 2 weeks. GAD-7 assessments were administered at baseline, after infusion 3, and at post-treatment follow-up. The Sheehan Disability Scale (SDS) and Work Productivity and Activity Impairment Questionnaire (WPAI) was used to measure functional impairment depending on the timing of the patient visiting the CTRCE. The SDS and WPAI assessed functional impairment in work/school and social and familial aspects of life on a scale of 0–10. These scales were assessed at baseline and after all ketamine infusions were completed. Data was collected directly at the point of care and entered into Research Electronic Data Capture at a baseline interview before treatment, at each subsequent infusion, and at post-treatment follow-up with a staff psychiatrist. Demographic data were collected at baseline during patient recruitment to the study.

Baseline vital signs, including heart rate and blood pressure were recorded prior to administration of IV ketamine. Blood pressure and heart rate were also evaluated throughout infusions every 15 min until 20 min after completion. Patients were monitored for dissociative symptoms throughout each infusion and 20 min post-treatment. Dissociative symptoms were scored using the modified six-item Clinician Administered Dissociative States Scales (CADSS) (see study protocol below). Any adverse symptoms such as nausea, headaches, or dizziness were recorded and assessed based on a scale of 1–4, from absent to severe.

### Study protocol

The CADSS were used to measure the severity of dissociative symptoms right after each IV ketamine infusion. A 6-item version of the CADSS was administered instead of the standard 23-item version to more efficiently assess symptoms of dissociation from ketamine (Rodrigues et al., 2021). The 6-item version of CADSS used questions 1, 2, 6, 7, 15, and 22 of the CADSS 23-item assessment. The reduced version (CADSS-6) strongly correlated to standard CADSS scores while reducing patient fatigue and time commitment. It also created a more targeted questionnaire for IV ketamine-induced dissociative symptoms instead of the original purpose of the CADSS, which is to assess dissociation in post-traumatic stress disorder (Rodrigues et al., 2021). A score of ⩾3 the CADSS-6 indicates the presence of dissociative symptoms.

This retrospective cohort study used a matched design based on baseline data where TAY CRTCE IV ketamine patients were matched with a control group of GA (aged 30–60; GA) patients to assess the difference in outcomes between age groups. This age range was chosen to be distinctly separate from TAY patients while avoiding the geriatric group (⩾65), where TRD may differ neurobiologically (Medeiros de Frota Ribeiro and Riva-Posse, 2017). The adult control group was created to have the same sex ratio (1:1), matched primary diagnosis, mean degree of treatment resistance (number of antidepressant treatments attempted), and initial depression severity (QIDS-SR16 baseline score). There was a tolerance of ±3 and 1 for the degree of treatment resistance and initial depressive symptom severity, respectively. Given the limited sample of available TAY patients, any patient with baseline data available was included in this study and matched with a GA patient even if data from subsequent infusions was not available.

### Data collection and analysis

The primary outcome of this retrospective analysis is the change in the severity of depressive symptoms measured using scores of the QIDS-SR16. Secondary outcomes were changes in the severity of anxiety symptoms and degree of functional impairment, assessed using the GAD-7 and SDS, respectively. Our analysis used a linear mixed-effects model to evaluate group differences between TAY (18–25) and GA patients (30–60). Several outcomes were assessed in this model: QIDS-SR16, QIDS-SR16 SI, GAD-7, SDS/WPAI work, family and social scores, as well as CADSS-6. Outcomes were assessed by the main effect of infusion and group, and an interaction of the two. Alpha was set to 0.05 and pairwise comparisons were conducted across infusions using the Bonferroni method to correct for multiple comparisons. Response to treatment was categorized by the percentage of reduction in QIDS-SR16 scores. Partial eta squared (η^2^) was used to evaluate effect size. Those with a ⩾25% reduction were classified as partial responders, and a complete response was a ⩾50% reduction. Any patient with a total QIDS-SR16 score ⩽5 was in remission from depression symptoms at that time.

## Results

### Baseline characteristics

In total, 54 patients were classified as transitional age; however, 2 patients were excluded from analysis as they lacked baseline total QIDS-SR16 scores. Of these patients, 48 patients were diagnosed with MDD, whereas the other 4 were diagnosed with bipolar depression. Following four infusions, 32 (61.5%) patients completed post-treatment follow-up clinical scales to assess symptom severity. Of note, completion of scales in the clinic is optional, leading to the significant amount of missing data (52 to 37 (TAY) and 38 (GA) participants from baseline to post-treatment follow-up). Other demographic data including mean age and body mass index, as well as matched variables of sex, diagnosis, and past antidepressants are presented in Table 1.

**Table 1.** Demographic data (*n* = 52) and SD for TAY (18–25) and GA (30–60) patients at baseline before treatment.

Characteristic	TAY (18–25)			GA (30–60)		
	Total	%	SD	Total	%	SD
Male	25	48.1	N/A	25	48.1	N/A
Female	27	51.9	N/A	27	51.9	N/A
Mean age (years)	22.04	N/A	1.74	45.04	N/A	8.80
Mean BMI (kg/m^2^)	24.39	N/A	6.07	27.88	N/A	5.09
Primary diagnosis of MDD	48	92.3	N/A	48	92.3	N/A
Primary diagnosis of BD	4	7.7	N/A	4	7.7	N/A
Mean # of past antidepressants	5.27	N/A	2.77	5.42	N/A	2.72
Mean # of current antidepressants	1.23	N/A	0.97	1.69	N/A	1.99
Previously treated with TMS and/or ECT	7	13.5	N/A	23	44.2	N/A

BMI: body mass index; SD: standard deviation; TAY: transitional age youth; GA: general adult; MDD: major depressive disorder; BD: bipolar disorder; TMS: transcranial magnetic stimulation; ECT: electroconvulsive therapy; N/A: not applicable.

### Depressive symptom severity

**Figure 1.**
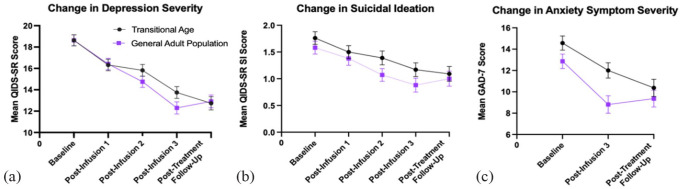
Plots of mean (±SE) QIDS-SR16 total (a), QIDS-SR16 suicidality (b), and GAD-7 total (c) scores baseline to post-treatment assessment in TAY and GA patients. SE: standard error; QIDS-SR16: Quick Inventory of Depressive Symptomatology Self-Report 16-item; GAD-7: Generalized Anxiety Disorder 7-item; TAY: transitional age youth.

There was a significant (*p* < 0.001) main effect of infusion on subsequent QIDS-SR16 total scores across both groups. (Figure 1 and Table 3). However, the main effect of the group (*p* = 0.402) and interaction between the group and infusion (*p* = 0.242) were found to be not significant indicating no significant differences in changes in depressive symptoms between TAY and GA patients. TAY patients experienced a 5.71-point drop in total QIDS-SR16 score, compared to a mean of 5.89-point drop in GA patients. Bonferonni adjusted pairwise comparisons indicate significantly lower total QIDS-SR16 scores from baseline to each infusion. The only infusion interval with insignificant changes in depression severity was from post-infusion 3 to follow-up (*p* = 1.000). There was a medium effect size for the main effect of infusion (η^2^ = 0.322).

At follow-up approximately 1 week after infusion 4, 9.4% of TAY patients scored as being in remission from depression based on total QIDS-SR16 score (Table 2). By comparison, GA patients had a remission rate of 8.6%. At follow-up, 25% of TAY patients had an antidepressant response (⩾50% reduction) and 18.8% had a partial response (⩾25% reduction). In GA patients, 31.4% had met response criteria and 8.6% had a partial response (Table 2).

**Table 2.** Categorical outcomes based on % reduction of total score QIDS-SR16 across all infusions in TAY and GA patients compared to baseline.

Response type	Post-infusion 1	Post-infusion 2	Post-infusion 3	Post-treatment
TAY				
Total (*N* =)	49	44	44	37
Non-response (⩽25% reduction)	39 (81.3)	39 (88.6)	23 (53.5)	15 (46.9)
Partial response (25–50% reduction)	7 (14.6)	3 (6.8)	15 (34.9)	6 (18.8)
Response (⩾50% reduction)	2 (4.2)	2 (4.5)	5 (11.6)	11 (34.4)
Remission (QIDS-SR16 of 5 or under)	1 (2.1)	0 (0)	1 (2.3)	3 (9.4)
GA				
Total (*N* =)	46	49	39	38
Non-response (⩽25% reduction)	33 (73.3)	32 (66.7)	16 (42.1)	18 (51.4)
Partial response (25–50% reduction)	9 (20.0)	8 (16.7)	12 (31.6)	3 (8.6)
Response (⩾50% reduction)	3 (6.7)	8 (16.7)	10 (26.3)	14 (40.0)
Remission (QIDS-SR16 of 5 or under)	1 (2.2)	1 (2.1)	1 (2.6)	3 (8.6)

The categorical outcome is expressed as a total (*N*) and proportion of the group in parentheses (%).

QIDS-SR16: Quick Inventory of Depressive Symptomatology Self-Report 16-item; TAY: transitional age youth; GA: general adult.

**Table 3.** Omnibus of statistical analysis of clinical measurements between both TAY and GA patient groups.

Interaction	Clinical outcome	*F* statistic	*p* Value	Effect size (η^2^)
Main effect of infusion	Total QIDS-SR16	*F* (4, 314) = 37.187	<0.001	0.322
	QIDS-SR16 SI	*F* (4, 318) = 11.328	<0.001	0.125
	GAD-7	*F* (2, 138) = 25.781	<0.001	0.271
	SDS/WPAI work	*F* (4, 67) = 3.250	0.017	0.162
	SDS/WPAI social	*F* (2, 147) = 15.063	<0.001	0.170
	SDS/WPAI family	*F* (2, 131) = 11.686	<0.001	0.152
	CADSS	*F* (3, 275) = 3.692	0.012	0.039
Main effect of group	Total QIDS-SR16	*F* (1, 121) = 0.707	0.402	<0.001
	QIDS-SR16 SI	*F* (1, 109) = 1.989	0.161	0.018
	GAD-7	*F* (1, 106) = 4.526	0.036	0.041
	SDS/WPAI work	*F* (1, 82) = 0.354	0.017	0.004
	SDS/WPAI social	*F* (1, 129) = 1.483	0.225	0.011
	SDS/WPAI family	*F* (1, 120) = 3.541	0.062	0.056
	CADSS	*F* (1, 109) = 0.571	0.451	0.005
Interaction effect of group and infusion	Total QIDS-SR16	*F* (4, 314) = 1.376	0.242	0.017
	QIDS-SR16 SI	*F* (4, 318) = 0.971	0.423	0.012
	GAD-7	*F* (2, 139) = 2.995	0.053	0.041
	SDS/WPAI work	*F* (2. 88) = 0.400	0.671	0.009
	SDS/WPAI social	*F* (2, 148) = 0.790	0.456	0.011
	SDS/WPAI family	*F* (2, 131) = 0.017	0.983	<0.001
	CADSS	*F* (3, 275) = 0.121	0.947	0.001

QIDS-SR16: Quick Inventory of Depressive Symptomatology Self-Report 16-item; SI: suicidal ideation; TAY: transitional age youth; GA: general adult; GAD-7: Generalized Anxiety Disorder 7-item; SDS: Sheehan Disability Scale; WPAI: Work Productivity and Activity Impairment Questionnaire; CADSS: Clinician Administered Dissociative States Scales.

### Suicidal ideation

Both TAY and GA patients experienced a main effect of infusion on suicidality as measured by QIDS-SR16 SI category (*p* < 0.001). There was not a significant main effect of group (*p* = 0.161), as well as an insignificant interaction between group and infusions (*p* = 0.423), indicating comparable reduction in suicidal thoughts. TAY patients had a 0.67-point decrease in suicidality, while GA patients had an average decrease of 0.58 points. Pairwise comparisons showed significant decrease in suicidality from baseline to post-infusion 2, post-infusion 3, and follow-up (*p* < 0.001). There was also a significant decrease in suicidality from post-infusion 1 to post-infusion 3 (*p* < 0.001). Only a small effect size for the main effect of infusion existed for suicidality symptoms (η^2^ = 0.125). However, 48 of 104 total patients had a SI score of 1 or less, including 20 of 52 TAY patients. There may be a ceiling effect in terms of the efficacy of IV ketamine on suicidality considering that 38% of TAY and 54% of GA patients had low or no SI at baseline.

### Generalized anxiety symptoms

**Figure 2.**
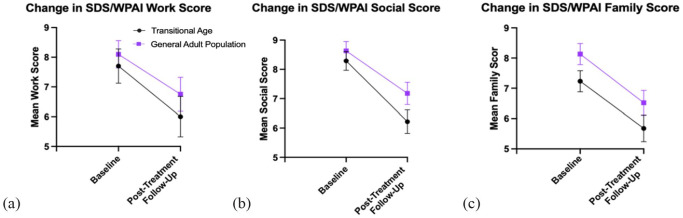
Plots of mean (±SE) SDS/WPAI work (a), social (b), and family (c) scores at baseline and post-treatment assessment in TAY and GA patients. SE: standard error; SDS: Sheehan Disability Scale; WPAI: Work Productivity and Activity Impairment Questionnaire; TAY: transitional age youth; GA: general adult.

TAY and GA patients experienced a significant main effect of infusion on anxiety symptoms (*p* < 0.001) measured by the GAD-7. There was a significant effect of age group (*p* = 0.036) on reduction in GAD-7 score. The effect of interaction between group and infusion (*p* = 0.053) on GAD-7 score was found to be insignificant, although only marginally for a confidence level of 95%. TAY patients had a mean decrease of anxiety symptom severity of 3.85 points from baseline to follow-up. GA had a mean reduction of 3.50 points. Pairwise comparisons revealed a significant reduction in GAD-7 score from baseline to post-infusion 3 and follow-up (*p* < 0.001), but not from post-infusion 3 to follow-up (*p* = 0.900). The effect size was medium for the main effect of infusion (η^2^ = 0.271) and was not significant for the effect of group or interaction (Figure 2).

### Functionality

A significant main effect of infusion existed for the SDS/WPAI assessment of work (*p* = 0.017), social (*p* < 0.001) and family (*p* < 0.001) functioning. There was also a main effect of the group on work (*p* = 0.017), but this effect was insignificant for social (*p* = 0.225), and family (*p* = 0.062) functioning. As with previous outcomes, there was also no significant interaction effect of group and infusion for work (*p* = 0.671), social (*p* = 0.456) and family (*p* = 0.983) assessments. Similarly, to the main effect of infusion, pairwise comparisons indicated a significant improvement in functioning from baseline to follow-up for work (*p* = 0.025), social (*p* < 0.001), and family (*p* < 0.001) contexts. Only small effect sizes were found for the main effect of infusion in work (η^2^ = 0.162), social (η^2^ = 0.170), and family (η^2^ = 0.152) functioning.

TAY patients had a mean drop in SDS/WPAI work score from baseline to follow-up of 1.70 points, compared to 1.35 points in GA patients. The mean decrease in social score was 2.07 points for TAY, whereas GA had a mean decrease of 1.45 points. Lastly, TAY patients saw a mean decrease in family score of 1.55 points. GA patients had a mean decrease in family score 1.61 points from baseline to post-treatment follow-up.

### Safety and tolerability

**Figure 3.**
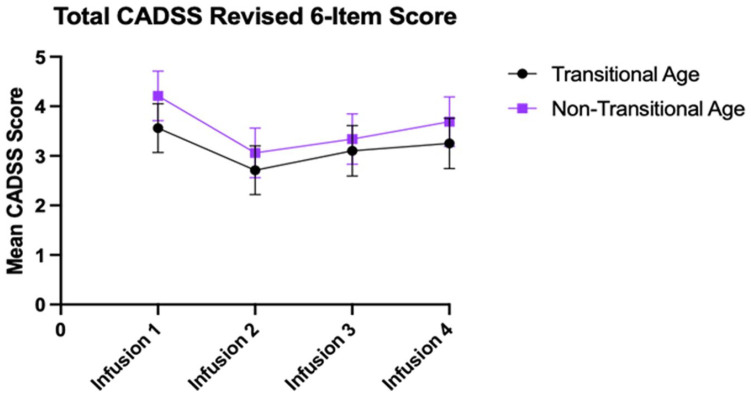
Plot of mean (±SE) CADSS-6 score at each infusion in TAY and GA patients. SE: standard error; CADSS: Clinician Administered Dissociative States Scales; TAY: transitional age youth; GA: general adult.

The main effect of infusion on CADSS-6 score was found to be significant (*p* = 0.012), despite a slight increase in CADSS-6 score from post-infusion 2 to follow-up in both groups. However, the main group effect (*p* = 0.451) and effect interaction (*p* = 0.947) on CADSS-6 score were also not significant. On average, both TAY and GA patients had the highest CADSS-6 score at infusion 1. The lowest mean CADSS-6 score occurred at infusion 2 (TAY = 2.71, GA = 3.06), with subsequent CADSS-6 scores worsening from thereon, although not significantly. The mean difference in CADSS-6 score from infusion 1 to infusion 4 was 0.30 points in TAY patients, and was 0.52 for GA patients (Figure 3 and Table 4).

**Table 4.** Reporting of specific adverse events during and after (20–30 min) any infusion in TAY and GA patients as a total (*n*) and percentage (%).

Adverse effect			During infusion		After infusion	
	TAY total	GA total	TAY *n* (%)	GA *n* (%)	TAY *n* (%)	GA *n* (%)
Nausea	38	24	19 (50.0)	9 (37.5)	13 (37.5)	9 (34.2)
Vomiting	38	22	3 (7.9)	1 (4.5)	2 (4.5)	1 (5.3)
Dizziness	36	23	30 (83.3)	16 (69.6)	28 (26.1)	6 (26.1)
Headache	37	22	5 (13.5)	3 (13.6)	10 (26.1)	6 (27.8)
Double vision	38	23	23 (60.5)	8 (34.8)	14 (31.8)	7 (37.8)
Blurred vision	38	22	27 (71.1)	11 (50.0)	13 (45.5)	10 (35.1)
Drowsiness	38	22	33 (86.8)	20 (90.9)	31 (81.8)	18 (81.6)
Confusion	38	23	27 (71.1)	17 (73.9)	16 (47.8)	11 (42.1)
Jerky muscle movement	37	23	16 (43.2)	2 (8.7)	2 (4.5)	1 (5.3)

TAY: transitional age youth; GA: general adult.

## Discussion

While repeated IV ketamine has been found to reduce symptoms of depression, suicidality, and anxiety in the GA TRD population, there is relatively less evidence with respect to ketamine in TAY patients specifically. The analysis presented herein found no significant difference in treatment outcomes between TAY and GA patients receiving IV ketamine for TRD. Both groups experienced a main effect of infusion (*p* < 0.05) on all clinical outcomes. On average, both groups of patients saw a decrease in depression severity based on QIDS-SR16 score with >5 point reduction indicative of a clinically meaningful improvement.

TAY patients in this study experienced a significant decrease in symptoms of SI from baseline to after repeated ketamine infusions that has been reported in the GA TRD population (McIntyre et al., 2021; Serafini et al., 2014). While the anti-suicidal response in TAY and GA patients was not significantly different, a trend appears to exist where GA patients may have anti-suicidal response after infusions 2 and 3. By post-treatment follow-up both patient groups reported similar levels of improvement in symptoms of SI. More investigation into comparing the duration required for an anti-suicidal response to IV ketamine should be conducted with a larger sample size to improve statistical power and using assessments that evaluate SI in more detail. In comparison to monoaminergic antidepressants such as selective serotonin reuptake inhibitors (SSRIs), IV ketamine appears to provide antidepressant effects more rapidly in TAY (Murphy et al., 2021). SSRIs can have anti-suicidal effects in TAY patients with depression, but provide these effects after a longer duration compared to IV ketamine (Murphy et al., 2021).

There was a significant difference in anxiety symptoms severity between groups as measured by GAD-7. Baseline data suggests that TAY patients with TRD may have greater anxiety symptoms severity than older TRD patients. A trend existed where GA patients had a more rapid decrease in anxiety symptoms after receiving IV ketamine. A least square mean (LSM) analysis was conducted to evaluate whether or not GA patients had a more rapid response to ketamine with regards to anxiety symptoms at post-infusion 3. However, this LSM analysis showed that the difference in response based on anxiety severity at post-infusion 3 was not statistically significant (*p* = 0.299).

It is possible that due to a smaller sample size of GA patients with GAD-7 scores at post-infusion 3 (*n* = 26), there is simply not enough statistical power to detect a significant difference. Despite this statistical insignificance, the use of IV ketamine on anxiety symptoms for the TAY population warrants further investigation. On average, TAY patients were more anxious at every time point compared to GA patients, although a significant difference was not detected. Considering that TAY patients were matched for identical sex and primary diagnosis, and similar treatment resistance and baseline depression severity, baseline data suggests that initial anxiety symptom severity differences may be related to age. As well, with a main effect of group on GAD-7 score, there is a justifiable need to further explore the difference in treating anxiety in TAY patients with TRD.

TAY patients possess a similar baseline QIDS-SR16 score compared to a larger (*n* = 190) sample of TRD patients (baseline QIDS-SR16 of 18.54) who have been treated at the CRTCE, but was not adjusted for comparison by age (McIntyre et al., 2020). This indicates that baseline depressive symptoms severity is similar across ages of those who received IV ketamine treatments at CRTCE for TRD. Similarly, TAY patients had a similar degree of treatment resistance based on the mean number of past antidepressants taken (*p* = 0.213) compared to a larger population of TRD patients at the CRTCE previously published (McIntyre et al., 2020). Based on previous literature using failed treatments to assess resistance, the degree of TRD is comparable in TAY patients to the larger TRD population.

While this study reports no serious adverse events during and after ketamine infusions, the milder side effects calling for moderate treatment were still quite prevalent. It should be noted that the tolerability of IV ketamine has been found to improve over subsequent infusions and reported side effects lasted for a short duration after infusion, and did not limit future treatments (Rodrigues et al., 2020). As well, this study cannot assess the long-term safety of IV ketamine infusions as the duration of treatment at the CRTCE is typically around 2 weeks with a total of four infusions. Maintenance phases of ketamine infusions every 2–6 weeks after an initial 3 weeks of twice-weekly treatments have been found to be well-tolerated in GA depression patients (Sakurai et al., 2020). However, transient effects of dissociation and nausea were still common (Sakurai et al., 2020). More investigation into the long-term tolerability and safety of ketamine infusions for depression is warranted in both TAY and GA patient populations.

There are limitations to this analysis relating to its methodology and data collection. Firstly, this investigation was retrospective, and lacked a proper control group that occurred in real time. In addition, many patients are missing data given the voluntary nature of the scales completed. The comparison group was created to match accurately on baseline characteristics, but did not exclude those who did not complete all scales after baseline. While a comparison group was used, the study is still limited by not using a placebo control group for comparison instead. Lastly, a more thorough investigation of anxiety severity between age groups could have been conducted if not for the need to control baseline depression severity (QIDS-SR16) to create a comparison group. As a result, only 26 GA patients had GAD-7 evaluations at post-infusion 3. GAD-7 could also have been assessed at all time points to better display the treatment efficacy over time. Despite these limitations, this analysis presents findings in an understudied population of TAY individuals with TRD in a real-world setting.

## Conclusion

In summary, repeated doses of IV ketamine have similar effects on reducing symptom severity of depression, anxiety, suicidality, and functional impairment in TAY patients as observed in GA patients. There was a small difference between groups in terms of anxiety symptom response, but the trend of significantly decreased anxiety compared to baseline was found in both groups. Additionally, ketamine infusions are similarly well-tolerated and safe when used in TAY patients compared to the GA group. Therefore, IV ketamine merits further investigation for treating TRD in individuals that are between 18 and 25 years of age. Considering minimal adverse outcomes associated with both the TAY and GA populations, the efficacy and safety of ketamine presents significant opportunities for improvement in mood disorder care. Randomized control trials should be conducted to further evaluate the efficacy and safety of repeated IV ketamine for TRD in TAY specifically.
